# Dietary Dried Jujube Fruit Powder (DJFP) Supplementation Improves Growth Performance, Antioxidant Stability, and Meat Composition in Broilers

**DOI:** 10.3390/foods12071463

**Published:** 2023-03-29

**Authors:** Chao Yang, Xijin Zhu, Wenyu Liu, Jie Huang, Zhijun Xie, Farong Yang, Li Zhang, Yuming Wei

**Affiliations:** 1 College of Food Science and Engineering, Gansu Agricultural University, Lanzhou 730070, China; 2 Animal Husbandry, Pasture and Green Agriculture Institute, Gansu Academy of Agricultural Sciences, Lanzhou 730070, China

**Keywords:** broiler, oxidative stress, dried jujube fruit powder, growth performance, antioxidants, antioxidant stability, meat quality, amino acid profile, fatty acid profile

## Abstract

Nowadays, broiler production is faced with great challenges due to intensive culture modes, and chickens are more susceptible to oxidative stress. Consequently, synthetic antioxidants have been used to reduce this process, but their use has shown potential health risks. Thus, the use of natural ingredients has been suggested as a strategy to prevent oxidative stress. This study investigated how dietary dried jujube fruit powder (DJFP) supplementation influences the growth performance, antioxidant stability, meat composition, and nutritional quality of Cobb broilers. A total of 360 unsexed broilers (1-day-old) were randomly assigned to treatments that varied in DJFP levels: a basal diet without DJFP (control) and diets supplemented with 50 g/kg DJFP (P1), 100 g/kg DJFP (P2), and 150 g/kg DJFP (P3), with 9 replicates per treatment (90 broilers/treatment or 10 broilers/replicate). The results demonstrated improvement in the growth performance of broilers in terms of body weight (BW), body weight gain (WG), average daily body weight gain (ADG), average daily feed intake (ADFI), and feed conversion ratio (FCR) following dietary DJFP supplementation. In addition, the antioxidant stabilities in the DJFP-treated broilers were improved and inhibited the production of lipid oxidation products compared with the control, with those in the P2 group showing the most marked effect. Moreover, dietary DJFP supplementation significantly increased (*p* < 0.05) the activity of antioxidant enzymes in broilers. Furthermore, the breast meat of the broilers displayed an increased protein content with a simultaneous reduction in the fat content after DJFP treatment (*p* < 0.05). Essential amino acid levels were higher in the DJFP-supplemented groups (*p* < 0.05). The sum of saturated fatty acids was lower, and that of monounsaturated fatty acids (MUFAs) and the polyunsaturated fatty acid/saturated fatty acid ratio (PUFA/SFA) were higher in the DJFP-supplemented groups (*p* < 0.05). Together, these results indicate that up to 100 g/kg of dietary DJFP supplementation can enhance the growth performance and antioxidant capacity, meat composition, and amino acid and fatty acid composition in broiler breast meat. In conclusion, dietary DJFP supplementation is a healthy alternative to the use of synthetic antioxidants in broiler production, especially in regions rich in jujube resources.

## 1. Introduction

Chicken, one of the most commonly consumed animal protein sources, has low fat, high protein, and high unsaturated fatty acid levels [1,2]. With the ever-growing demand for chicken meat, intensive farming methods have been introduced into broiler production to enhance productivity [3]. Unfortunately, these intensive practices tend to negatively affect the physiological balance in farmed broilers, which induces oxidative stress [4] and compromises broiler performance and meat quality.

To protect against excess ROS accumulation and oxidative stress in broilers, antioxidants are one of the most important defense means to delay reactions by scavenging chain-propagating peroxyl radicals [5]. Synthetic antioxidants that prevent the formulation of free radicals are commonly used [6]. However, in recent years, there has been a growing interest in natural antioxidants, often derived from waste materials or by-products, due to potential safety concerns associated with synthetic antioxidants [7]. Exploring effective dietary sources, such as *Ginkgo biloba* extract, marigold extract, dried olive pulp, and jujube, could be interesting and rewarding [8,9]. *Ziziphus jujuba* Mill, a commonly known round and sour fruit, has been used for medicinal purposes for more than 2000 years [10]. There is general agreement that the antioxidant activities of plants are mostly associated with polysaccharides, polyphenols, and flavonoids with potent radical scavenging capabilities [11]. The jujube fruit is rich in these compounds that exhibit a high antioxidant capacity and alleviate oxidative stress in animals [12]. Mechanistically, the antioxidant mechanism of the jujube involves multiple pathways. First, the phenolic compounds in the jujube directly neutralize free radicals by donating electrons or hydrogen atoms, thus preventing oxidative damage to cellular components such as lipids, proteins, and DNA. Additionally, flavonoids in the jujube modulate the activities of antioxidant enzymes, such as SOD, CAT, and GPX, leading to an elevated scavenging of reactive oxygen species (ROS) and maintenance of redox homeostasis. Moreover, triterpenoids have been shown to activate the Nrf2-ARE signaling pathway, which regulates the expression of antioxidant and detoxification genes in the body [13]. This results in an increased production of antioxidant enzymes, leading to an enhanced antioxidant defense. These compounds may act synergistically to confer various bioactivities to the jujube [14]. Over the past 20 years, global jujube production has more than doubled. China is the largest producer of the jujube, contributing to over 90% of the world’s total production. Correspondingly, a great deal of waste is generated because some products are deemed unfit for sale and are discarded, causing significant destruction of plant resources [15]. The use of the jujube as an animal dietary supplementation is a promising application under investigation. The addition of a moderate level (150 g/kg) of jujube powder to feed led to an amelioration of the growth performance and meat quality of male Jinlan cashmere goats through a feeding experiment that persisted for 50 days [16]. Sami et al. reported that a supplementation of feed with 50% crushed date pit enhanced the growth performance and meat quality merits of Naimi lambs through a three-month feeding trial [17]. An analogous pattern was observed in aquatic products, wherein supplementing the diet of common carp with 0.5% jujube extract resulted in improved growth performance during an eight-week feeding trial [18].

Supplementing antioxidants in broiler feed may be a practical strategy to prevent damage to the body caused by oxidative stress, and consumers are increasingly inclined towards products containing antioxidants from natural sources compared to synthetic antioxidants. The jujube presents a potential natural source of antioxidants. Importantly, jujube fruits are richer in active constituents and are cheap and easily acquirable due to their huge reserve compared with other natural antioxidants [16]. However, the potential use of the jujube as a feed supplement in broilers’ diets has not been fully explored and the results of its use remain inconclusive. Therefore, it is hypothesized that dietary dried jujube fruit powder (DJFP) supplementation could be developed as a good unconventional feedstuff for broiler feed formulation. The results of the present study may serve as a theoretical basis for broiler industry development, contribute clarity to the potential applications of the jujube in poultry production, and may also offer a valuable reference for the future planning of the jujube by-product industrial chain.

## 2. Materials and Methods

### 2.1. Dried Jujube Fruit Powder (DJFP) Preparation

**Figure 1:**
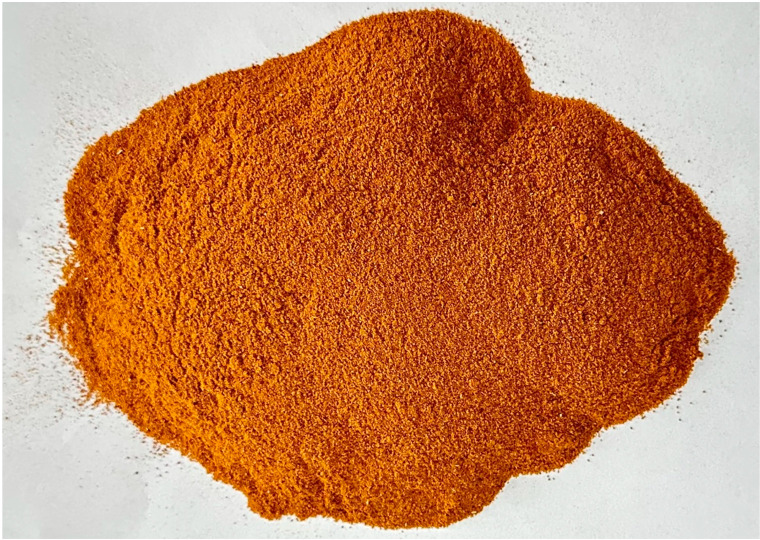
Dried jujube fruit powder.

Jujubes fruits were obtained from Qinfengtan Jujube Garden, Minqin County, Wuwei City, and Gansu Province. Guided by an expert, the waste jujubes were separated into a jujube automatic grading machine (Lekong Automation and Technology Co. Ltd., Zhucheng, Shandong, China). Subsequently, the waste jujube samples were taken back to the laboratory, then they were carefully washed with running water and dried for 8 h at 80 °C in a draft stove. The dried fruits were then ground and sieved using a jujube mill and 200 μm mesh, respectively (Figure 1). The obtained DJFP was packed in polyethylene plastic bags, sealed, labeled, and stored until further use.

### 2.2. Study Design, Treatment, and Management

This study was conducted at a poultry research farm following approval from the Faculty Animal Policy and Welfare Committee of Gansu Agricultural University (approval no. GSAU-Eth-AST-2021-001).

The study was carried out at poultry research farm sites at the Gansu Academy of Agricultural Sciences in Xiangruixin Village, Tianzhu County, Gansu Province, Gansu Province, in the period during September to December 2022. Thorough disinfection and sterilization of the chicken houses were performed before the start of the experiment. The range of DJFP dosage used in this study was determined through pre-experiments and reference to previous studies where DJFP was added to quail feed and goats’ diets. These dosages were selected as they did not cause any adverse reactions in broilers. White-feathered broilers are the most prevalent species in the intensive poultry management system, as they have a higher growth rate, muscle yield, and feed efficiency than traditional broilers. Therefore, white-feathered broilers were selected as subjects for this study. Three-hundred and sixty 1-d-old specific pathogen-free healthy Cobb 500 broilers (45.00 ± 1.27 g) were weighed and randomly divided into four groups that were fed corn–soybean meal diets supplemented with 0, 50 (P1), 100 (P2), and 150 g/kg DJFP (P3), respectively. The experimental design was a completely randomized design (CRD). The broilers were distributed in 36 cages, with 9 cages in each group, with 10 unsexed chickens housed per cage (length 100 cm, width 100 cm, and height 60 cm) at a stocking density of 10 birds/m^2^. Moreover, vaccination programs and veterinary care were implemented under the guidance of an expert veterinarian, as per the broiler husbandry practice. Birds had access to water and feed ad libitum throughout the feeding period.

Table 1 displays the ingredients and nutrition compositions of the experimental diets, formulated such that they met or exceeded the nutritional requirements of the National Research Council (NRC). The feeding period lasted for 42 days, and the composition was analyzed according to the standard procedures of the AOAC. Metabolic energy was calculated according to the NRC. For the first week of rearing, the temperature in the cages was maintained at 35 ± 0.5 °C and then gradually reduced after 7 days to 23 °C at a rate of 3 °C every 2 days. This temperature was maintained until the feeding program termination. The relative humidity was maintained at 50%.

### 2.3. Sample Collection Procedures

After 42 days of feeding, two chickens from each cage (body weight (BW) around the average weight) were randomly selected after 12 h of starvation. Hence, 18 birds were selected from each feeding group, and 72 animals (across 4 groups) were sacrificed via cervical dislocation. After de-feathering, samples of the breast tissue were collected and then the samples were sorted by treatment group. After the harvest, all broiler carcasses were chilled with static ice water (0–2 °C) in metal containers. Subsequently, breast (boneless and skinless) muscles were manually deboned from the carcass. A total of 72 whole breasts were placed into individually labeled Ziploc bags. To ensure consistency, the whole breast was divided into right and left parts. Breast samples were then individually vacuum-packaged in vacuum pouches and frozen at −80 °C until proximate analysis determinations could be performed. The breast halves corresponding to the right side were bagged, vacuum-packaged, and frozen (−80 °C) for biochemical analysis.

### 2.4. Proximate Composition of DJFP

#### 2.4.1. Chemical Composition

The chemical composition of the DJFP was examined in terms of crude protein (Kjeldahl method), ether extract (Soxhlet extraction method), crude fiber (Weende method), and crude ash (muffle furnace at 600 °C) contents according to Official Methods of Analysis by the AOAC. Neutral and acid detergent fibers were analyzed by an Ankom 200 fiber analyzer. After an aqua regia digestion phase, the calcium and phosphorus contents of the DJFP were determined by inductively coupled plasma and optical emission spectroscopy. 

#### 2.4.2. Total Phenolic

The determination of total phenolic compounds was carried out using the Folin–Ciocalteu reagent method. First, 0.5 L of distilled water and 125 µL of folinphenol were mixed with a 125 µL phenolic compounds solution. The mixtures reacted simultaneously for 6 min, and 1.25 mL Na_2_CO_3_ solution (7%) was followed by 1 mL of distilled water. The mixture was kept at room temperature in the dark for 90 min. The control was prepared by replacing the sample with methanol. Finally, the FOD was measured from 765 nm. The content of total polyphenols was presented as mg gallic acid equivalent GAE/100 g DW [19].

#### 2.4.3. Total Flavonoid

First, 100 µL of a sample extraction solution and 200 µL of the 5% NaNO_2_ solution were added to test tubes, respectively. After 6 min, the addition of 200 µL of the 5% NaNO_2_ solution. After 6 min of reaction time, followed by 2 mL of 4% NaOH solution, the mixture was kept in the dark at room temperature for 1.5 h, and the OD values (510 nm) were measured. Total flavonoid contents were calculated using a standard rutin curve and are shown as mg rutin equivalent (RE)/100 g DW [20].

#### 2.4.4. Tannin

First, 0.1 g of jujube flour was mixed with 4 mL of 1% HCl-methanol and extracted by using a tube mixer for 1 h at room temperature followed by collecting the supernatant after centrifuging at 1000× *g* for 10 min. A known volume of sample extract was made up to 250 μL by adding 1% HCl-methanol, and 1.25 mL of freshly prepared vanillin-HCl reagent was added as well. The sample blank was prepared by adding 4% HCl methanol. The samples were allowed to rest for 20 min at room temperature, and the OD was read at 500 nm against the sample blank, using catechin as standard. The tannin content was expressed as mg catechin equivalent (CE)/100 g of the sample [21].

### 2.5. Growth Performance Measurement

The BWs of each bird were determined using a digital balance (accuracy: 0.01 g, Ningbo Ji Ming Weighing Equipment Co., Ningbo, China) every evening throughout the experimental period, and the leftover feed was weighed and resupplied with the fixed diet. In addition, at the end of the rearing, 30 broiler chickens were randomly selected from each group of birds, 3 from each repetition. The body weight (WG), average daily feed intake (ADFI), daily weight gain (ADG), and feed conversion ratio (FCR) of the broilers following feeding program termination [22]. The WG, ADFI, ADG, and FCR were calculated as follows:

(1) WG=final BW−initial BW

(2) $$\mathrm{ADFI}=\frac{\mathrm{total}\;\mathrm{feed}\;\mathrm{intake}}{\mathrm{experimental}\;\mathrm{days}}$$

(3) $$\mathrm{ADG}=\frac{\mathrm{final}\;\mathrm{BW}-\mathrm{initial}\;\mathrm{BW}\;}{\mathrm{experimental}\;\mathrm{days}}$$

(4) $$\mathrm{FCR}=\frac{\mathrm{feed}\;\mathrm{intake}\;}{\mathrm{weight}\;\mathrm{gain}}$$

### 2.6. Preparation of Myofibrillar Protein

Myofibrillar proteins were prepared as described by Xiong et al. [23]. Briefly, meat samples (1 g) were added to a 4× volume (*v*/*w*) of phosphate buffer. The samples were homogenized and centrifuged at 2500× *g*, 4 °C for 15 min. After discarding the supernatant, phosphate-buffered saline (PBS) was added and additional centrifugation was performed. The pellets were then washed twice with a 4× volume (*v*/*w*) of extraction buffer. To remove the connective tissues, the homogenate was filtered through a 100-micron nylon mesh (Shanghai Hai Liang Filter Cloth Co., Ltd., Shanghai, China). Finally, the myofibrillar protein was obtained.

### 2.7. ABTS Free Radical-Scavenging Activity

The ABTS free radical scavenging activity was determined as previously described by Erel [24] after some adjustments. An ABTS stock was produced by mixing 50 mL of ABTS with potassium persulphate (200 mL, 70 mM). The stock was incubated in the dark for 15 h before use. It was then diluted with PBS to obtain an absorbance of 0.700 ± 0.030 at 734 nm. The diluted ABTS solution was mixed with 20 µL of the aqueous supernatants and allowed to stand for six minutes before the absorbances were read at 734 nm against PBS as the blank control. The ABTS scavenging activity in the meat was determined using the following formula:

(5) $$\mathrm{Scavenging}\;\mathrm{activity}\;(\%)=\left[\frac{A_{\mathrm{control}}-A_{\mathrm{test}}}{A_{\mathrm{control}}}\right]\times 100\%$$

where A_control_ represents the control absorbance value, and A_test_ represents the sample absorbance value.

### 2.8. Reactive Oxygen Species (ROS)

The ROS level was determined according to the method of Zhang et al. [25] with minor modifications. In brief, 2′,7′-dichlorodihydrofluorescein diacetate was used for the measurement of ROS. The meat samples were homogenized in an ice-cold homogenizing buffer at 10,000× *g* for 1 min, followed by centrifugation at 3900× *g* for 15 min. The protein concentration in the supernatant was calculated using the Biuret method. Subsequently, 100 µL of the supernatant was incubated with 100  µL of the buffer solution for 30 min at 37 °C. The fluorescence intensity was measured at 450 nm on an M200 Multiskan Spectrum system (TECAN, Atlanta, GA, USA).

### 2.9. Carbonyl Content

The 2,4-dinitrophenyl hydrazine (DNPH) method was used to estimate the carbonyl content in samples [26]. Diluted myofibrillar protein samples (5 mg/mL) were treated with DNPH at 37 °C in the dark for 60 min (one vortex every 15 min), and their absorbance was measured at 370 nm. The carbonyl content was calculated from a molar absorption coefficient of 2200 M^−1^·cm^−1^ and expressed as nmol carbonyl/mg proteins.

### 2.10. Measurement of Lipid Oxidation

The modified Folch procedure was used to extract total lipids and determine oxidative stability. The minced meat sample (5 g) was homogenized with a chloroform/methanol mixture (2:1, *v*/*v*). The resulting homogenate was then filtered using a separation funnel and a 0.88% KCl aqueous solution was added. After allowing the sample solution to rest for 20 h, the lower organic layer was collected and evaporated to constant weight at room temperature. The lipid extract was obtained by subtracting the final weight of the collector glass from its initial weight [4]. 

The primary oxidation product. The Peroxide value (PV) was determined using the ferric thiocyanate method and expressed as milliequivalents of oxygen molecule per kg of lipids (Meq O_2_/kg). To the lipid extract sample (0.1 g), 9.9 mL of chloroform/methanol (7:3, *v/v*) solution was added and vortexed. Subsequently, 50 μL of 10 mM xylenol orange solution and 50 μL FeCl_2_ solution (1000 mg/kg) were added to the mixture, which was then left to rest for 5 min at room temperature. A spectrophotometer measured the absorptivity at 560 nm. The spectrophotometric procedure was used to determine the value of conjugated dienes (CDs) and trienes (CTs) using a lipid extract sample dissolved in 2,2,4-trimethylpentane (iso-octane), and the absorbance values of the sample solution at 233 nm (CDs) and 268 nm (CTs) were assayed.

Secondary oxidation products. *P*-anisidine values were measured using an acidic method that depends on the reaction between anisidine and the aldehydes present in the lipid extract samples. The lipid extract was dissolved in iso-octane, and the absorbance of the solution was determined at 350 nm. Next, the *p*-anisidine reagent was introduced into the cuvette, then left in the dark for 10 min, and a new spectrum was recorded. TBARS levels were determined as previously described by Wang et al. [27]. First, meat samples (5 g) were chopped and homogenized in 40 mL of a 7.5% (*w*/*v*) TCA solution containing 0.1% (*w*/*v*) EDTA. The mixture was then filtered, and 2 mL of the filtrate was treated with 2 mL of 80 mM thiobarbituric acid. The same volume of deionized water was used as a control. The experiment was set for 90 min, and absorbance was measured at the 532 nm wavelength. Malondialdehyde (MDA) was used as a standard, and the TBARS value was expressed as MDA equivalents.

### 2.11. Analysis of Antioxidant Enzyme

The assay contents of superoxide dismutase (SOD), catalase (CAT), and glutathione peroxidase (GPX), were quantified using corresponding commercial kits (Nanjing Jiancheng Bioengineering Institute, Nanjing, Jiangsu, China), as per the manufacturer’s instructions.

### 2.12. Measurement of T-AOC

The total antioxidant capacity (T-AOC) was measured using a commercial kit according to the manufacturer’s instructions (Nanjing Jiancheng Bioengineering Institute, Jiancheng, Nanjing, Jiangsu, China). The absorbance values were measured using a microplate reader and the T-AOC was expressed as U/mg pro.

### 2.13. Chemical Composition

The moisture, crude fat, and crude ash contents of samples were analyzed according to the AOAC method 950.46B, AOAC method 960.39, and AOAC method 920.153, respectively. Crude protein concentration was assessed using the Kjeldahl method from AOAC method 981.10.

### 2.14. Analysis of Amino Acid Composition

A Hitachi L-8900 system (Hitachi, Tokyo, Japan) was used to analyze the amino acid composition of breast meat according to the method of Tian et al. [28]. The meat was ground using liquid nitrogen in a mortar and pestle, and 1 g samples were hydrolyzed for 22–24 h in 10 mL of 6 M HCl with a thermostatic drier at 110 °C. The liquid was transferred to 50 mL volumetric flasks and diluted at a constant volume using 0.02 M HCl. The solution was transferred to a clean glass dish and evaporated to dryness under a steady stream in a 65 °C water bath. The mixture was dissolved in 2 mL of 0.02 M HCl and filtered through a 0.22 µm filter. Amino acid separation was performed at a flow rate of 0.40 mL/min using a protein hydrolysate analysis column. Standard amino acid profiles were used for comparison.

### 2.15. Fatty Acid Profiles

The fatty acid composition of breast meat samples was analyzed using the method of Jung et al. [29] on an Agilent 7890A gas chromatograph equipped with a flame ionization detector and a DB-23 capillary column (60 m × 0.25 mm × 0.25 μm). 

### 2.16. Statistical Analysis

Data were analyzed by one-way analysis of variance (ANOVA) using SPSS 17.0 software (SPSS Inc., Chicago, IL, USA, 2008). Each cage (replicate) was considered an experimental unit. Differences between groups were determined by Duncan’s multiple range test and a *p*-value < 0.05 indicated a significant difference. Curve estimation including linear and quadratic responses was made by assessing the orthogonal polynomial contrasts.

## 3. Results

### 3.1. Proximate Composition of DJFP

The chemical composition and bioactive constituents of the DJFP are presented in Table 2. In general, jujube fruits vary in chemical composition depending on the cultivar, origin, climate, and harvesting time. The data in this study revealed that the DJFP contained 7.75% crude protein, 1.31% ether extract, 11.41% crude fiber, and 2.69% crude ash. Phenolics are biologically active secondary plant metabolites that serve as antioxidants. Furthermore, the analysis showed that the DJFP had a content of approximately 4.02 g/kg total polyphenols, 0.44 g/kg total flavonoids, and 0.78 g/kg total tannins, which agrees well with other studies [30]. There were greater concentrations of total phenols and flavonoids in the DJFP, which might reflect on the methods used for improving poultry growth as well as the serum biochemical parameters.

### 3.2. Growth Performance 

Growth performance is one of the most objective indicators reflecting broiler growth [31]. Here, it was found that dietary DJFP supplementation resulted in a marked improvement in the growth performance of broilers (Table 3). It was noted that the broilers in the P2 treatment showed the highest BW, WG, and ADG (*p* < 0.01). Accordingly, the ADFI values in the P2 and P3 groups were substantially elevated compared with the control, and there was a marked increase in the FCR of the broilers in the P1 group compared with the control and P2 groups (*p* < 0.05). In contrast, the mortality rate was higher for the control and P2 groups than for the P1 and P3 groups (*p* < 0.01). Furthermore, there were linear and quadratic increases in both WG and ADFI with DJFP supplementation (*p* < 0.05), while the BW and ADG values increased significantly (*p* < 0.05) in a linear fashion with DJFP supplementation.

### 3.3. Antioxidant Stability 

**Figure 2:**
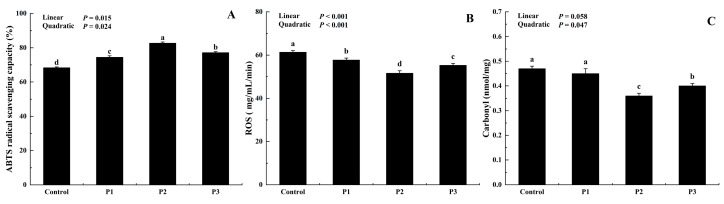
Effect of dietary dried jujube fruit powder (DJFP) supplementation on the antioxidant capacity of the broilers. (**A**) Effect of dietary DJFP supplementation on the ABTS radical-scavenging capacity of the broilers. (**B**) Effect of dietary DJFP supplementation on the ROS content of the broilers. (**C**) Effect of dietary DJFP supplementation on the carbonyl content of the broilers. Control, P1, P2, and P3 diets contained 0, 50, 100, and 150 g/kg DJFP in the diet, respectively. ROS, reactive oxygen species; ^a–d^ Values with different letters above the column mean significant difference (*p* < 0.05).

The effect of dietary DJFP on the antioxidant status of broilers was investigated by monitoring indicators of oxidative stress, including the ABTS radical-scavenging capacity, ROS, and carbonyl content levels (Figure 2). Significantly increased ABTS radical scavenging was observed after the addition of DJFP compared to the control (*p* < 0.01). Moreover, DJFP effectively reduced ROS production (*p* < 0.01). The carbonyl content, a measure of protein oxidation, decreased significantly in the P2 and P3 treatments when compared to their control and P1 counterparts (*p* < 0.05). We found that the ABTS radical-scavenging capacity in the sample increased with a linear (*p* < 0.05) and quadratic (*p* < 0.05) response to DJFP supplementation, and significantly decreased ROS contents with linear (*p* < 0.001) and quadratic (*p* < 0.001) responses were observed in the DJFP-supplemented groups while both the carbonyl contents decreased with a quadratic (*p* < 0.05) response with DJFP supplementation. Overall, the comparative analysis showed that the P2 group had the highest degree of antioxidant stability.

### 3.4. Lipid Oxidation

**Figure 3:**
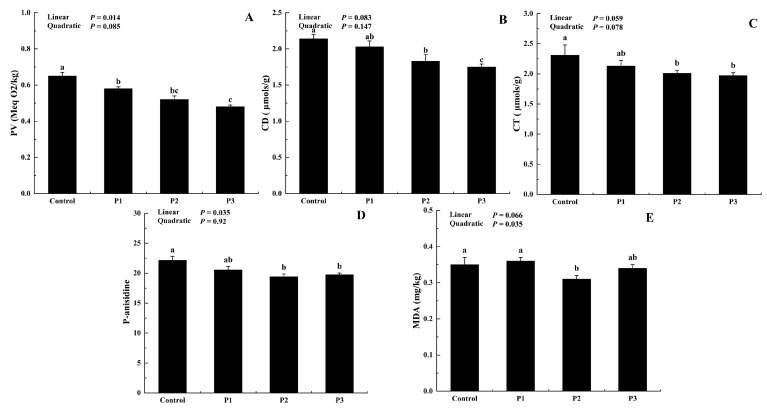
Effect of dietary dried jujube fruit powder (DJFP) supplementation on the products of lipid oxidation of the broilers. (**A**) Effect of dietary DJFP supplementation on the Peroxide value of the broilers. (**B**) Effect of dietary DJFP supplementation the conjugated dienes value of the broilers. (**C**) Effect of dietary DJFP supplementation on the conjugated trienes value of the broilers. (**D**) Effect of dietary DJFP on the Para anisidine of the broilers. (**E**) Effect of dietary DJFP supplementation on the MDA content of the broilers. Control, P1, P2, and P3 diets contained 0, 50, 100, and 150 g/kg of DJFP in the diet, respectively. MDA, malondialdehyde. ^a–c^ Values with different letters above the column mean significant difference (*p* < 0.05).

The stability of lipid oxidation depends on the balance between antioxidant and pro-oxidant components [32]. Figure 3 demonstrates the positive influence of dietary DJFP supplementation on lipid peroxidation parameters. Compared with the control group, primary oxidation products formed, including PV, CD, and CT, were decreased when DJFP was included in diets (*p* < 0.05). In addition, the PV decrease appeared to be linear over the addition of DJFP (*p* < 0.05). When the DJFP addition was 150 g/kg, lipid oxidation was reduced significantly with the best inhibition effect. Moreover, supplementing diets with DJFP led to a marked reduction in secondary lipid oxidation products (*p* < 0.05). Among them, the best effect was observed with 100 g/kg of DJFP, as indicated by lower values of MDA (0.31 mg/kg) and *p*-anisidine (19.41) compared with the control. These achievements proved the significant efficacy of the antioxidant compounds of DJFP included in the diets in delaying the peroxidation process of broilers.

### 3.5. Antioxidant Enzyme Analysis

**Figure 4:**
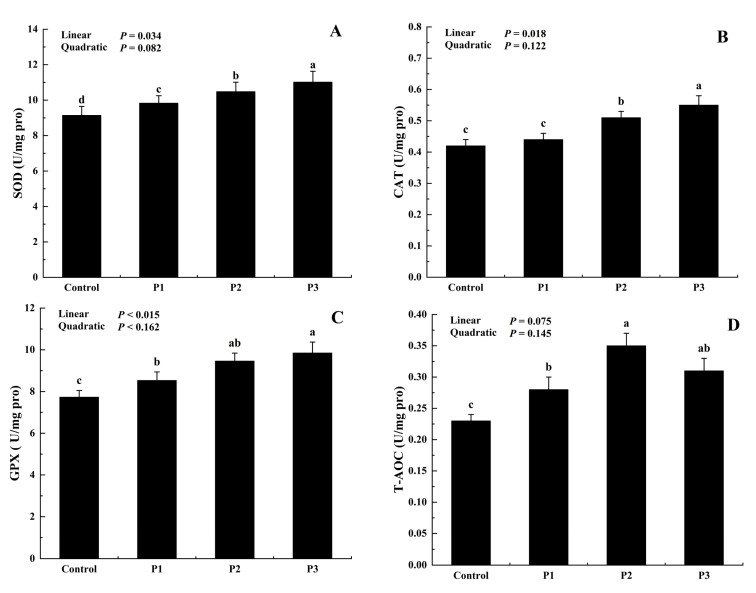
Effect of dietary dried jujube fruit powder (DJFP) supplementation on the antioxidant enzyme activity of the broilers. (**A**) Effect of dietary DJFP supplementation on the SOD. (**B**) Effect of dietary DJFP supplementation on the CAT of the broilers. (**C**) Effect of dietary DJFP supplementation on GPX of the broilers. (**D**) Effect of dietary DJFP supplementation on the T-AOC of the broilers. Control, P1, P2, and P3 diets contained 0, 50, 100, and 150 g/kg of DJFP in the diet, respectively. ^a–d^ Values with different letters above the column mean significant difference (*p* < 0.05).

Antioxidant enzymes are the determining factor in maintaining redox homeostasis and inhibit oxidative stress by suppressing lipid peroxidation and augmenting antioxidant enzymes toward the control [33]. As shown in Figure 4, the addition of DJFP resulted in a notable increase in SOD, CAT, and GPX activities in comparison to the control (*p* < 0.05). Among them, SOD, CAT, and GPX activity reached the highest levels in the P3 treatment while T-AOC obtained the maximum in the P2. In addition, the SOD, CAT, and GPX activity in the sample all enhanced with a linear (*p* < 0.05) response with DJFP supplementation.

### 3.6. Chemical Composition

**Figure 5:**
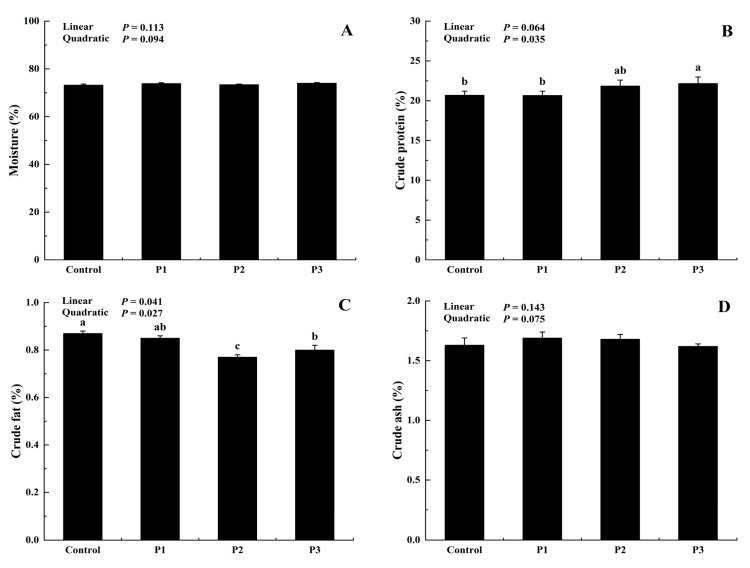
Effect of dietary dried jujube fruit powder (DJFP) supplementation on breast meat composition of broilers. (**A**) Effect of dietary DJFP supplementation on the moisture content of the broilers. (**B**) Effect of dietary DJFP supplementation on the crude protein content of the broilers. (**C**) Effect of dietary DJFP supplementation on the crude fat content of the broilers. (**D**) Effect of dietary DJFP supplementation on the crude ash content of the broilers. Control, P1, P2, and P3 diets contained 0, 50, 100, and 150 g/kg DJFP in the diet, respectively. ^a–c^ Values with different letters above the column mean significant difference (*p* < 0.05).

The chemical composition of breast meat from the broilers after supplementation with different levels of DJFP is shown in Figure 5. The addition of DJFP did not significantly affect the moisture and crude ash contents of chicken breasts (*p* > 0.05). Nevertheless, it was found that DJFP supplementation markedly elevated the crude protein content (*p* < 0.05), with the addition of 150 g/kg showing the best effect with values of 22.17% and a quadratic (*p*< 0.05) response in the crude protein content to DJFP supplementation. In contrast, the crude fat content in the sample decreased with a linear (*p* < 0.05) and quadratic (*p* < 0.05) response to DJFP supplementation, and a significant decrease in crude fat content among DJFP-supplemented groups was observed (*p* < 0.05).

### 3.7. Amino Acid Composition

Apart from their nutritional importance, amino acids affect the palatability and aroma of meat through the production of flavor substances via the Maillard reactions [34]. As shown in Table 4, 17 amino acids were identified in all treatments. The highest contents of amino acids were observed in the P2 and P3 groups, followed by the P1 group, and the lowest level was reported for the control group (*p* < 0.05). At the highest level of DJFP supplementation (150 mg/kg), the contents of Met (1.59), Thr (7.25), and His (1.81) reached maximum values. Moreover, chickens supplemented with 100 mg/kg of DJFP showed marked increases in the Phe, Tyr, and Cys contents. Specifically, the level was elevated by 7.4% following the addition of 100 g/kg of DJFP compared to the control. The overall rise in the amino acid contents was attributed to the concomitant rise of individual amino acids, i.e., Phe, Met, Leu, His, Ala, Tyr, and Cys (*p* < 0.05). Meanwhile, linear (*p* < 0.05) and quadratic (*p* < 0.05) increases in the Leu and His contents, respectively, were observed with the inclusion of DJFP in the diet, and the Phe and Cys contents increased with a quadratic (*p* < 0.05) response with DJFP supplementation. Finally, there was a linear (*p* < 0.05) enhancement in essential amino acid and non-essential amino acid contents and a quadratic (*p* < 0.05) elevation in the amino acid level in response to increasing DJFP supplementation.

### 3.8. Fatty Acid Profiles 

Fatty acids are critical for energy maintenance and as cellular nutrients. The highest content detected was for oleic acid (C18:1), followed by linoleic acid (C18:2n-6) (Table 5). Together, these molecules accounted for approximately 70% of the fatty acid content, consistent with previous findings [35]. The levels of SFAs, particularly pentadecanoic acid (C15:0), arachidic acid (C20:0) (*p* < 0.01), and tricosanoic acid (C23:0), were reduced after DJFP supplementation (*p* < 0.05). The DJFP-supplemented groups also exhibited a marked increase in the levels of partial sums of monounsaturated fatty acids (MUFAs) (*p* < 0.05), which reflected the trend of myristoleic acid (C14:1) and palmitoleic acid (C16:1); among them, the P2 group provided the most pronounced effect. Furthermore, DJFP supplementation increased the partial sums of PUFA content, with significant enhancements observed in α-linolenic acid (C18:3n-3), Gamma-linolenic acid (C18:3n-6), and eicosapentaenoic acid (C20:5n-3) (*p* < 0.05). The ω-6 PUFA content was improved gradually in P2 and P3 treatments compared with the control counterpart (*p* < 0.05). However, no significant treatment effect was observed on the ω-3 PUFA levels of breast meat, resulting in a significant DJFP-concentration-related decrease in the ω-6: ω-3 PUFA ratio. Specifically, pentadecanoic acid (C15:0) showed a linear decrease with incremental DJFP inclusion (*p* < 0.05). However, there was a linear (*p* < 0.05) elevation in the contents of myristoleic acid (C14:1) and palmitoleic acid (C16:1), and docosahexaenoic acid (C22:6n-3) showed a quadratic (*p* < 0.05) increase with DJFP supplementation. Lastly, supplementation of DJFP resulted in a linear (*p* < 0.01) and quadratic (*p* < 0.05) increase in the unsaturated fatty acid (USFA)/SFA and PUFA/SFA ratios with the incremental supplementation of DJFP.

## 4. Discussion

There has been a rise in the incidence of oxidative stress in broilers due to the increasing demands of intensive production [36]. Persistent exposure to stress decreases the feed intake, growth performance [37], and meat quality of animals [38]. Recently, feed interventions with natural antioxidants have gained popularity to protect broilers against oxidative stress through the scavenging of free radicals [39]. In particular, the jujube contains significant concentrations of flavonoids and polyphenols, which exhibit antioxidant properties. Furthermore, jujube fruits are more suitable as feed supplements than other natural antioxidants. Thus, in the current experiment, we evaluated the growth performance, antioxidant stability, and meat composition of broilers fed with DJFP supplementation.

The quality of dietary supplements has a direct influence on broiler performance and forage value [40]. In the current study, the chemical analysis of DJFP was consistent with the experimental results of similar studies [41]. In particular, the DJFP used contained less than 2% fat. In general, the jujube should be considered a low-fat dietary supplement [42]. Moreover, it contains active compounds, such as polyphenols and flavonoids, that possess antioxidant, immune-boosting, and antitumor properties [43,44]. Its abundant supply, relatively low price, and various health benefits have encouraged its use in animal feeds.

In the present study, DJFP supplementation to the diet markedly enhanced the growth performance of broiler chickens. This situation is in agreement with a previous study reporting that the inclusion of 1% jujube in the basal diet positively influenced both the live weight and daily live weight of quails during a feeding trial of 38 days [45]. A supplementation of 0.1 g/kg of essential oils was found to improve the growth performance of broiler chicks through a trial that lasted for 35 days [46]. Similar findings have been observed in other species. Waste jujube meal has been reported to be an optimal and cheap feedstock when used at levels of up to 150 g/kg dietary supplementation in 14-month-old male Jinlan cashmere goats, where it showed positive effects on growth and carcass characteristics in a 50-day trial [16]. A dietary supplementation of 0.5% jujube fruit extracts was also found to increase the performance parameters of common carp during an eight-week feeding trial [18]. We then analyzed the cause of this phenomenon. The mechanisms through which DJFP promotes the growth performance of broilers are probably multiple. Firstly, a variety of aromatic substances, such as acids and hydrocarbons, may be produced during the jujube drying process [16]. The palatability of feed is the main factor that affects feed intake, and it is expected that DJFP inclusion in the diet can enhance the palatability of feed due to its taste to enhance feed intake in broilers with a consequent improvement in growth performance [47]. Secondly, flavonoids in jujubes are likely to contribute to improved growth performance through their beneficial effects on the gut functions of broilers [48]. Thirdly, the beneficial effects of flavonoids from jujubes on the growth of broilers may be associated with the upregulation of growth hormones and hepatic growth hormone receptors. This phenomenon may increase the levels of insulin-like growth factor 1 and ultimately promote animal growth.

In this study, DJFP was found to improve the antioxidant status of broiler chickens. This is likely because the jujube fruit contains abundant polyphenols, such as protocatechuic, gallic, and chlorogenic acids, which are strongly associated with powerful antioxidant properties [49]. Additionally, the strong antioxidant effects of polyphenols could also be attributed to their function as reducing agents [50]. Polyphenols reduce free radicals and convert them to a more stable state while scavenging radicals that are potentially harmful to the human body. Thus, they indirectly potentiate the antioxidant capacity of meat [51]. Further, these findings are in line with the results that the addition of 9% jujube pulp improves the antioxidant activities of goat milk yogurt [52]. Therefore, DJFP can serve as a potential antioxidant source for poultry.

Generally, the oxidative stability of meat is determined by analyzing three primary oxidation products (PV, CD, and CT) and two secondary oxidation products (*p*-anisidine and MDA) [4]. The findings from the analysis of oxidative stability in breast meat demonstrated that the rate of lipid oxidation was influenced by the presence and amount of DJFP in the diet, revealing a dose-dependent effect of DJFP on the oxidative stability of meat. This might be because jujubes are rich in antioxidants, such as phenolic compounds, flavonoids, and carotenoids, which can scavenge free radicals and prevent lipid oxidation [30]. These antioxidants work by donating electrons to stabilize free radicals and inhibit chain reactions that lead to lipid oxidation.

The principal defense mechanisms against oxidative stress are the enzymatic and non-enzymatic antioxidant systems, which have a crucial function in sustaining the redox equilibrium [53]. SOD is a critical antioxidant enzyme that can transform the superoxide anion into a less harmful compound, namely hydrogen peroxide (H_2_O_2_) [54]. CAT and GPX are capable of breaking down H_2_O_2_ into water. [55]. T-AOC, an all-inclusive index, can indicate the overall antioxidant status [56]. As a result, T-AOC, SOD, CAT, and GPX are frequently employed as reliable markers to evaluate the antioxidant capacity of animals. Our study demonstrated that dietary DJFP supplementation significantly increased the activity of SOD, CAT, GPX, and T-AOC in breast muscle. Similar results were reported that a diet supplemented with jujube of 1% improves antioxidant enzyme activities in the muscle of Japanese quails [45]. The positive effects of jujubes on muscle antioxidant capacity were also demonstrated in rainbow trout [57].

A substantial elevation in the crude protein content was observed in broilers fed with the DJFP-supplemented diet (Figure 5). This could be due to the presence of abundant cyclic adenosine monophosphate (cAMP) [58], which can promote or prolong protein synthesis in vivo by stimulating the secretion of growth hormones [59]. Furthermore, skeletal muscle proteolysis could be inhibited by the activation of the cAMP-dependent pathway [60]. In this trial, the observed reduction in crude fat accumulation could likely be attributed to the presence of abundant flavonoids in the jujube fruit (Table 2). Many recent studies have shown that flavonoid intake can reduce body fat deposition in animals [61,62]. In particular, dietary flavonoids inhibit 3T3-L1 preadipocyte adipogenesis through mitotic clonal expansion suppression and adipogenic transcriptional cascade regulation [63,64]. The conclusions of the current study are consistent with these observations. Diets supplemented with 0.7% and 1.0% fermented *Ginkgo biloba* leaves were shown to reduce the deposition of abdominal fat in Arbor Acres broiler chicks during a 42-day feeding trial [65]. A similar finding was reported in an earlier study that, when Jinlan cashmere goats were fed 150 g/kg of Chinese jujube fruits in the diet, the protein content of the meat was substantially enhanced by 10% during a 50-day feeding trial [16].

Intensively farmed chickens often suffer from oxidative stress, due to the increased generation of free radicals and reduced antioxidant defense [66]. The direct oxidation of amino acid side chains causes loss and generates several carbonyl products (primary carbonylation) [67]. The contents of Phe, Met, Leu, His, Ala, Tyr, and Cys were markedly enhanced by DJFP supplementation (Table 4). The possible reasons behind this improved enhancement may be summarized into the following points. Firstly, jujubes contain a range of active antioxidant ingredients, such as alkaloids, terpenes, flavonoids, polysaccharides, phenols, and vitamins [68]. These active substances assist the body in scavenging excess free radicals and increase the antioxidant properties of the body, thereby inhibiting the occurrence of lipid and protein oxidation and thus indirectly influencing amino acid loss [69]. This observation is consistent with the findings of the antioxidant stability experiment (Figure 2). Some studies suggest that the jujube may stimulate protein synthesis by increasing the activity of mTOR, a key regulator of protein synthesis in cells. By increasing protein synthesis, DJFP may help to increase the amino acid content of meat. Moreover, due to the high contents of carbohydrates in DJFP, some carbohydrate transport and metabolism-related genes have been proven to contribute to amino acid biosynthesis. These could indirectly compensate for the amino acid loss in breast meat by accelerating the biosynthesis of amino acids through the regulation of a series of amino acid metabolism related genes, such as Phe, and Tyr [70]. Thus, DJFP can be added to animal feeds during intensive broiler rearing to improve meat quality and achieve multiple benefits.

SFAs have been associated with the development of coronary heart disease due to their hypercholesterolemic properties. Decreasing the levels of dietary SFAs and increasing those of MUFAs in meat may reduce the threat of cardiovascular disease and promote human health [71]. In the current research, dietary DJFP supplementation considerably reduced the pentadecanoic acid (C15:0), arachidic acid (C20:0), and tricosanoic acid (C23:0) content in breast meat (Table 5). This may be because the addition of DJFP to the feed increases the expression of Δ9-desaturase and in turn elevates Δ9-desaturase activity, which are enzymes that play a key role in the conversion of SFA to MUFA. These enzymes introduce a double bond at the ninth carbon position of a fatty acid chain, thereby converting it from a saturated to a monounsaturated fatty acid. Once the double bond is introduced, the fatty acid can be further modified by other enzymes to PUFA [72]. Furthermore, the jujube contains various bioactive compounds, which can scavenge free radicals and reduce oxidative stress in animals, contributing to the improvement of lipid metabolism and fatty acid composition in meat. DJFP also contains high levels of dietary fiber, which may improve gut health and nutrient absorption, leading to a better metabolism and utilization of dietary fats. Moreover, DJFP may enhance the expression of genes involved in fatty acid oxidation and reduce the expression of genes involved in fatty acid synthesis, leading to a shift toward USFA synthesis. Overall, the exact mechanisms by which DJFP affects the conversion of saturated to unsaturated fatty acids are complex and may involve multiple pathways, including antioxidant activity, gut health, and gene regulation. The majority of the increase in USFAs was myristoleic acid (C14:1), palmitoleic acid (C16:1), α-linolenic acid (C18:3n-3), gamma-linolenic acid (C18:3n-6), and Eicosapentaenoic acid (C20:5n-3). A higher dietary intake of ω-3 polyunsaturated fatty acids (PUFAs), including alpha-linolenic acid (ALA), eicosapentaenoic acid (EPA), and docosahexaenoic acid (DHA), has been well documented to provide health benefits, particularly in the control of cardio and cerebrovascular diseases in humans. In normal human diets, a consistent increase in the ω-6: ω-3 PUFA ratio has been associated with an increased incidence of cardiovascular disease [73]. Here, the ω-6: ω-3 PUFA ratio in broilers decreased with dietary supplementations of DJFP, suggesting that the fatty acid profiles were improved with better health indices. USFAs are recognized as important components of the human diet, and they are converted to eicosanoids, such as leukotrienes and prostaglandins, to regulate both immune and cardiovascular function [74]. MUFA and PUFA levels were notably higher in the experimental P2 and P3 groups (e.g., myristoleic acid, palmitoleic acid, and docosahexaenoic acid), which may be due to the conversion of SFAs to USFAs. Moreover, the PUFA/SFA ratio can be used in evaluating the quality of fat. Decreased PUFA/SFA ratios may enhance the absorption of cholesterol, resulting in a greater accumulation of cholesterol in the liver and promoting hypercholesterolemia [75]. The experimental treatments P1, P2, and P3 increased the PUFA/SFA ratio, demonstrating that DJFP may have a favorable effect on the breast meat quality of broilers.

## 5. Conclusions

The current study demonstrated that dietary supplementation with DJFP not only increased the growth performance and antioxidant capacity of broiler chickens but also improved the chickens’ quality and muscle amino acid and fatty acid profiles, because (1) DJFP supplementation enhanced the BW, WG, ADG, ADFI, and FCR of the chickens; (2) it can enhance the ABTS radical-scavenging capacity of the chickens while lowering the ROS and carbonyl contents; (3) it inhibits the PV, CD, CT, Para anisidine, and MDA production of the broilers; (4) it improve chickens’ SOD, CAT, GPX, and T-AOC activity; (5) it could decrease the fat content and increase the protein content; (6) it could increase the essential amino acid levels in muscle; and (7) it has the ability to improve fatty acid profiles in broilers. The optimum inclusion level of DJFP was found to be 100 g/kg under these experimental conditions. Therefore, our results suggested that DJFP, as a natural antioxidant, may ameliorate oxidative stress and improve the growth performance and meat quality of chickens. The findings of this study may help us to further explore the possible mechanisms by which DJFP protects against oxidative stress and contribute clarity to the potential applications of DJFP in pig production.

## Figures and Tables

**Table 1:** Ingredients and chemical composition of the experimental diets.

Ingredients, %	1–21 Days				22–42 Days			
	Dried Jujube Fruit Powder, g/kg				Dried Jujube Fruit Powder, g/kg			
	0	50	100	150	0	50	100	150
Yellow maize	47.77	44.77	43.27	40.52	51.01	48.51	45.51	43.4
Dried jujube fruit powder	0	5.0	10.0	15.0	0	5.0	10.0	15.0
Soybean meal	31.3	29.3	28.8	27.55	28.3	27.3	26.2	24.6
Corn gluten meal	5.0	5.0	4.0	3.5	3.5	3.0	2.8	2.36
Cottonseed meal	7.43	7.43	6.13	5.93	8.42	7.92	7.42	6.82
Soybean oil	3.0	3.0	2.3	2.0	4.0	3.5	3.3	3.05
Limestone	1.52	1.52	1.52	1.52	1.31	1.31	1.31	1.31
Dicalcium phosphate	2.18	2.18	2.18	2.18	1.74	1.74	1.74	1.74
L-Lysine	0.39	0.39	0.39	0.39	0.33	0.33	0.33	0.33
DL-Methionine	0.16	0.16	0.16	0.16	0.14	0.14	0.14	0.14
Common salt	0.25	0.25	0.25	0.25	0.25	0.25	0.25	0.25
Vitamin–mineral premix ^1^	1.0	1.0	1.0	1.0	1.0	1.0	1.0	1.0
Calculated composition ^2^								
Metabolizable energy (kcal/kg)	2947.36	2953.64	3013.52	3085.43	3097.83	3137.21	3183.73	3225.63
Calcium, %	1.0	1.13	1.21	1.24	0.84	0.89	0.91	0.93
Available phosphorus, %	0.45	0.45	0.48	0.49	0.35	0.38	0.41	0.4
SID lysine, % ^3^	1.15	1.17	1.2	1.19	1.05	1.07	1.1	1.11
SID methionine, % ^3^	0.49	0.5	0.53	0.55	0.44	0.44	0.47	0.49
Analyzed composition								
Dry matter, %	86.64	86.95	87.38	86.73	85.73	86.15	86.66	86.63
Crude protein, %	21.02	21.04	20.86	20.83	19.43	19.34	19.19	19.15
Ether extract, %	3.63	3.62	3.58	3.55	3.52	3.48	3.44	3.47
Crude fiber, %	3.43	3.51	3.58	3.66	3.35	3.48	3.54	3.59
Crude ash, %	4.72	4.93	5.25	5.31	4.52	4.68	4.82	4.97
Nitrogen-free extract, %	53.84	53.85	54.11	53.38	54.91	55.17	55.67	55.45

^1^ Vitamin–mineral premix provided per kilogram of diet: 1300 IU of vitamin A, 3300 IU of vitamin D, 17.5 mg of vitamin E, 2 mg of thiamine, 8 mg of riboflavin, 10 mg of niacin, 60 mg of pantothenic acid, 3.50 mg of pyridoxine, 0.02 mg of cobalamin, 450 mg of choline, 0.06 mg of biotin, 80 mg of Fe, 8 mg of Cu, 60 mg of Mn, 50 mg of Zn, 0.45 mg of I, and 0.30 mg of Se. ^2^ Calculated according to National Research Council requirements. ^3^ Values for standardized ileal digestible (SID) lysine and methionine.

**Table 2:** Chemical composition and bioactive constituents of dried jujube powder (as-dry basis).

Chemical Composition		Bioactive Constituents	
Dry matter (%)	87.55	Total polyphenols ^1^ (g/kg)	4.02
Nitrogen-free extract (%)	64.39	Total flavonoids ^2^ (g/kg)	0.44
Crude protein (%)	7.75	Total tannins ^3^ (g/kg)	0.78
Ether extract (%)	1.31	cAMP ^4^ (g/kg)	2.54
Crude ash (%)	2.69	cGMP ^5^ (g/kg)	1.34
Crude fiber (%)	11.41	Lysine (g/100 g)	0.14
Neutral detergent fiber (%)	11.43	Threonine (g/100 g)	0.13
Acid detergent fiber (%)	9.12		
Calcium (%)	0.62		
Phosphorus (%)	0.25		

^1^ Result expressed as mg gallic acid equivalent/g of dry matter. ^2^ Result expressed as mg rutin equivalent/g of dry matter. ^3^ Result expressed as mg catechol equivalent/g of dry matter. ^4^ cAMP, cyclic adenosine monophosphate. ^5^ cGMP, cyclic monophosphate.

**Table 3:** Effect of dietary supplementation with dried jujube powder on growth performance of broilers.

Item	Treatment ^2^				SEM ^1^	*p*-Value	*p*-Value	
	Control	P1	P2	P3			Linear	Quadratic
BW, g	1688.3 ^d^	1772.25 ^c^	1885.55 ^a^	1864.31 ^b^	24.82	0.008	0.037	0.072
WG, g	1642.61 ^d^	1728.11 ^c^	1840.36 ^a^	1818.01 ^b^	22.57	0.006	0.042	0.028
ADG, g	39.11 ^d^	41.15 ^c^	43.82 ^a^	43.29 ^b^	0.59	<0.001	0.041	0.058
ADFI, g	84.35 ^c^	91.21 ^b^	94.95 ^a^	94.66 ^a,b^	2.17	0.005	0.032	0.046
FCR, g/g	2.16 ^b^	2.22 ^a^	2.17 ^b^	2.19 ^a,b^	0.02	0.034	0.084	0.075
Mortality, %	1.11 ^a^	0.00 ^b^	1.11 ^a^	0.00 ^b^	0.01	<0.001	0.084	0.063

^a–d^ within the same row with no common superscript show significant differences (*p* < 0.05). BW, body weight; WG, body weight gain; ADG, average daily body weight gain; ADFI, average daily feed intake; FCR, feed conversion ratio. ^1^ SEM: standard error of the mean. ^2^ Control, P1, P2, and P3 diets contained 0, 50, 100, and 150 g/kg of dried jujube fruit powder (DJFP) in the diet, respectively.

**Table 4:** Effect of dietary supplementation with dried jujube powder on the amino acid composition of broiler breast meat.

Item, g/100 g Pro	Treatment ^2^				SEM ^1^	*p*-Value	*p*-Value	
	Control	P1	P2	P3			Linear	Quadratic
**Essential**								
Lysine (Lys)	4.42	4.55	4.81	4.71	0.263	0.124	0.098	0.124
Phenylalanine (Phe)	4.96 ^c^	5.34 ^b^	5.71 ^a^	5.53 ^a,b^	0.244	0.038	0.085	0.043
Valine (Val)	1.66	1.71	1.84	1.77	0.158	0.197	0.335	0.074
Methionine (Met)	1.49 ^c^	1.53 ^b^	1.5 ^c^	1.59 ^a^	0.02	0.006	0.068	0.103
Isoleucine (Ile)	1.55	1.57	1.71	1.66	0.063	0.068	0.105	0.059
Leucine (Leu)	6.6 ^d^	6.92 ^c^	7.15 ^b^	7.25 ^a^	0.006	0.035	0.043	0.019
Threonine (Thr)	2.54	2.68	2.79	2.64	0.138	0.148	0.104	0.418
Arginine (Arg)	6.43	6.27	6.74	6.65	0.266	0.316	0.064	0.079
Histidine (His)	1.41 ^d^	1.72 ^b^	1.68 ^c^	1.81 ^a^	0.015	0.027	0.029	0.037
**Non-Essential**								
Aspartic acid (Asp)	6.65	6.94	7.32	7.18	0.364	0.112	0.083	0.112
Serine (Ser)	4.01	3.93	3.83	3.9	0.015	0.362	0.412	0.315
Glutamic acid (Glu)	11.43	11.68	12.14	11.65	0.512	0.425	0.057	0.068
Glycine (Gly)	2.87	3.03	2.92	3.09	0.038	0.141	0.312	0.279
Alanine (Ala)	4.83 ^b^	5.01 ^a^	4.8 ^b^	4.95 ^a,b^	0.062	0.034	0.075	0.068
Tyrosine (Tyr)	3.91 ^c^	4.16 ^b^	4.54 ^a^	4.47 ^a,b^	0.065	0.021	0.078	0.052
Cysteine (Cys)	0.17 ^d^	0.22 ^c^	0.29 ^a^	0.26 ^b^	0.006	0.043	0.074	0.038
Proline (Pro)	1.84	1.91	1.96	1.92	0.034	0.115	0.104	0.076
**Partial Sums of Amino Acid Groups**								
Essential	31.06 ^c^	32.29 ^b^	33.93 ^a^	33.61 ^a,b^	0.253	0.036	0.044	0.074
Non-essential	35.71 ^b^	36.88 ^a,b^	37.8 ^a^	37.42 ^a^	0.558	0.028	0.039	0.091
Amino acid	66.77 ^c^	69.17 ^b^	71.73 ^a^	71.03 ^a^	0.636	0.019	0.054	0.037

^a–d^ within the same row with no common superscript show significant differences (*p* < 0.05). ^1^ SEM: standard error of the mean. ^2^ Control, P1, P2, and P3 diets contained 0, 50, 100, and 150 g/kg of dried jujube fruit powder (DJFP) in the diet, respectively.

**Table 5:** Effect of dietary supplementation with dried jujube powder on the fatty acid composition of broiler breast meat.

Item, g/100 g Total Fatty Acid	Treatment ^2^				SEM ^1^	*p*-Value	*p*-Value	
	Control	P1	P2	P3			Linear	Quadratic
**SFA**								
Myristic acid (C14:0)	0.134	0.128	0.122	0.122	0.014	0.063	0.077	0.117
Pentadecanoic acid (C15:0)	0.514 ^a^	0.459 ^b^	0.426 ^c^	0.397 ^d^	0.015	0.005	0.034	0.224
Palmitic acid (C16:0)	7.204	7.012	7.306	7.023	0.196	0.214	0.257	0.122
Stearic acid (C18:0)	3.444	3.271	3.267	3.215	0.226	0.258	0.334	0.252
Arachidic acid (C20:0)	0.051 ^a^	0.049 ^a,b^	0.045 ^c^	0.047 ^b^	0.001	0.007	0.139	0.221
Behenic acid (C22:0)	0.062	0.057	0.061	0.063	0.002	0.102	0.178	0.208
Tricosanoic acid (C23:0)	4.112 ^a^	3.674 ^c^	3.882 ^b^	3.854 ^b^	0.257	0.026	0.463	0.083
Lignoceric acid (C24:0)	0.016	0.014	0.014	0.015	0.001	0.377	0.133	0.301
**MUFA**								
Myristoleic acid (C14:1)	0.086 ^b^	0.079 ^c^	0.084 ^b,c^	0.089 ^a^	0.002	0.006	0.035	0.085
Palmitoleic acid (C16:1)	0.664 ^c^	0.714 ^b^	0.768 ^a^	0.754 ^a,b^	0.013	0.036	0.033	0.025
Oleic acid (C18:1)	8.741	8.816	9.144	9.056	0.156	0.115	0.064	0.112
Gadoleic acid (C20:1)	0.441 ^a^	0.426 ^b^	0.434 ^a,b^	0.44 ^a^	0.012	0.028	0.442	0.183
Erucic acid (C22:1)	0.111	0.12	0.103	0.114	0.003	0.058	0.557	0.384
**PUFA**								
Linoleic acid (C18:2n-6c)	8.341	8.336	8.662	8.746	0.635	0.082	0.103	0.122
α-linolenic acid (C18:3n-3)	0.36 ^b,c^	0.353 ^c^	0.383 ^a^	0.367 ^b^	0.005	0.024	0.063	0.075
Gamma-linolenic acid (C18:3n-6)	0.044 ^c^	0.051 ^b^	0.055 ^a^	0.049 ^b,c^	0.003	0.005	0.071	0.083
Eicosatrienoic acid (C20:3n-6)	0.151	0.164	0.168	0.169	0.002	0.153	0.062	0.079
Arachidonic acid (C20:4n-6)	2.084	2.112	2.188	2.211	0.127	0.523	0.368	0.424
Eicosapentaenoic acid (C20:5n-3)	0.177 ^c^	0.203 ^b^	0.185 ^b,c^	0.23 ^a^	0.012	0.006	0.075	0.088
Docosahexaenoic acid (C22:6n-3)	0.411	0.446	0.427	0.451	0.021	0.082	0.113	0.092
**Partial sums of fatty acid ^1^**								
SFA	15.537	14.664	15.123	14.736	0.497	0.157	0.554	0.335
MUFA	10.043 ^b^	10.155 ^b^	10.533 ^a^	10.453 ^a,b^	0.179	0.038	0.072	0.083
PUFA	11.568	11.665	12.068	12.223	0.168	0.062	0.064	0.074
ω-3 PUFA	0.948	1.002	0.995	1.048	0.018	0.105	0.089	0.104
ω-6 PUFA	10.62 ^b^	10.663 ^b^	11.073 ^a,b^	11.175 ^a^	0.136	0.018	0.072	0.097
ω-6/ω-3 PUFA ratio	11.203 ^a^	10.642 ^b^	11.129 ^a,b^	10.663 ^b^	0.337	0.033	0.103	0.112
USFA/SFA	1.391 ^b^	1.488 ^a,b^	1.494 ^a^	1.539 ^a^	0.015	0.018	0.034	0.038
PUFA/SFA	0.745 ^c^	0.795 ^b^	0.798 ^b^	0.829 ^a^	0.006	0.006	0.043	0.035

^a–d^ within the same row with no common superscript show significant differences (*p* < 0.05). SFA (saturated fatty acid) = C14:0 + C15:0 + C16:0 + C18:0 + C20:0 + C22:0 + C23:0 + C24:0; MUFA (monounsaturated fatty acid) = C14:1 + C16:1 + C18:1 + C20:1 + C22:1; PUFA (polyunsaturated fatty acids) = C18:2n-6c + C18:3n-3 + C18:3n-6 + C20:3n-6 + C20:4n-6 + C20:5n-3 + C22:6n-3. USFA, unsaturated fatty acid. ω-3 PUFA = C18:3n-3 + C20:5n-3 + C22:6n-3; ω-6 PUFA = C18:2n-6c + C18:3n-6 + C20:3n-6 + C20:4n-6. ^1^ SEM: standard error of the mean. ^2^ Control, P1, P2, and P3 diets contained 0, 50, 100, and 150 g/kg of dried jujube fruit powder (DJFP) in the diet, respectively.

## Data Availability

The data used and/or analysed in this study are available from the corresponding author on reasonable request.
